# Detection and Characterization of Hepatitis E Virus Genotype 3 in Wastewater and Urban Surface Waters in Germany

**DOI:** 10.1007/s12560-020-09424-2

**Published:** 2020-03-14

**Authors:** Sophia Beyer, Regine Szewzyk, Regina Gnirss, Reimar Johne, Hans-Christoph Selinka

**Affiliations:** 1 Section II 1.4 Microbiological Risks, German Environment Agency (UBA), Corrensplatz 1, 14195 Berlin, Germany; 2 Berliner Wasserbetriebe (BWB), Cicerostr. 24, 10709 Berlin, Germany; 3 German Federal Institute for Risk Assessment (BfR), Max-Dohrn-Straße 8-10, 10589 Berlin, Germany

**Keywords:** Hepatitis E virus, Monitoring, Genotyping, Wastewater, Surface water, Combined sewer overflow

## Abstract

In highly populated areas, environmental surveillance of wastewater and surface waters is a key factor to control the circulation of viruses and risks for public health. Hepatitis E virus (HEV) genotype 3 is considered as an emerging pathogen in industrialized countries. Therefore, this study was carried out to determine the prevalence of HEV in environmental waters in urban and suburban regions in Germany. HEV was monitored in water samples using quantitative RT-PCR (RT-qPCR) and nested RT-PCR without or with virus concentration via polyethylene glycol precipitation or ultracentrifugation. By RT-qPCR, 84–100% of influent samples of wastewater treatment plants were positive for HEV RNA. Genotypes HEV-3c and 3f were identified in wastewater, with HEV-3c being the most prevalent genotype. These data correlate with subtypes identified earlier in patients from the same area. Comparison of wastewater influent and effluent samples revealed a reduction of HEV RNA of about 1 log_10_ during passage through wastewater treatment plants. In addition, combined sewer overflows (CSOs) after heavy rainfalls were shown to release HEV RNA into surface waters. About 75% of urban river samples taken during these CSO events were positive for HEV RNA by RT-qPCR. In contrast, under normal weather conditions, only around 30% of river samples and 15% of samples from a bathing water located at an urban river were positive for HEV. Median concentrations of HEV RNA of all tested samples at this bathing water were below the limit of detection.

## Introduction

Hepatitis E virus (HEV) is the causative agent of acute and chronic hepatitis in humans worldwide. A severe disease progression is possible with mortality rates around 1% (Pérez-Gracia et al. 2015). However, among pregnant women infected with HEV genotype 1, a higher incidence and severity was observed with mortality rates up to 30% (Clemente-Casares et al. 2016).

HEV is classified into four main human-pathogenic genotypes within the *Hepeviridae* family. Genotypes 1 and 2 infect only humans and are endemic in developing countries. Genotypes 3 and 4 are zoonotic and infect mainly humans, swine and wild boars (Pavio et al. 2017). Whereas genotype 4 is mainly restricted to Asia, in most industrialized countries genotype 3 is predominant (Clemente-Casares et al. 2003; Meng 2010; Dalton et al. 2014).

In Germany seroprevalences for HEV-specific antibodies of about 1% in children (Krumbholz et al. 2014) and about 15% among adults (Faber et al. 2018a) were reported. A continuous increase in the number of notified hepatitis E cases was recorded in Germany during the last years, most likely due to increased awareness. In 2018 about 3400 new hepatitis E cases were reported to the Robert Koch Institute (RKI 2019).

HEV is transmitted mainly through meat products of infected animals and faecally contaminated water. Transmission to humans through contaminated water is known for genotypes HEV-1 and HEV-2, mainly in developing countries (Fenaux et al. 2019).

Industrialization of a country decreases HEV risk related to HEV-1, but increases that related to HEV-3 and HEV-4, as observed in China (Sridhar et al. 2015).

So far, the role of water in the transmission of zoonotic HEV-3 has only been suspected (Fenaux et al. 2019). A recent study identified the occupational contact with wastewater as a risk factor associated with autochthonous hepatitis E in Germany, supporting that waterborne transmission of HEV-3 is possible (Faber et al. 2018b).

In developed countries human and animal hosts of HEV-3 may contaminate wastewater through their faeces. HEV particles can reach the environment and potentially contaminate surface waters. Thus, surface waters could be a source of HEV contamination for animals and humans (Fenaux et al. 2019).

Increasing HEV prevalence in industrialized countries is known since 1998. In Spain, HEV detection in urban sewage samples was reported (Pina et al. 1998), followed by reports from the Netherlands (Rutjes et al. 2009), Italy (La Rosa et al. 2010) and other countries. Most common detection methods are nested reverse transcription (RT) polymerase chain reaction (PCR) or quantitative RT-PCR (RT-qPCR) with or without prior virus concentration steps. In recent years, Italy, Norway and the UK have reported first investigations for a HEV surveillance in sewage (Idolo et al. 2013; Myrmel et al. 2015; Smith et al. 2016a; Alfonsi et al. 2018).

To the best of our knowledge there are no available studies on the presence of HEV in environmental waters in Germany. Therefore, this study was carried out to (1) investigate the HEV prevalence in environmental water samples, (2) to compare HEV concentration methods, and (3) to genotype detected HEV strains. Wastewater influent and effluent samples of urban and suburban wastewater treatment plants (WWTPs), surface waters from two rivers including a bathing water and conditions of combined sewer overflows (CSO) were investigated. For comparison of virus concentration techniques, PEG-precipitated samples, samples subjected to ultracentrifugation and samples without further virus concentrations were tested simultaneously. Genotyping was performed for further characterization of the detected HEV strains.

## Material and Methods

### Sampling

Samples of wastewater influents (after coarse grid removal) and wastewater effluents (secondary effluents, before UV treatment) of WWTPs were collected in the years 2014–2019 from central urban (WWTP 1) and suburban (WWTPs 2–4) areas of the cities of Berlin and Munich, Germany. Surface water samples were taken in the years 2016–2019 from two urban rivers at normal weather conditions (river 1 and river 2), as well as after heavy rainfall events with combined sewer overflows (river 1/CSO). CSO samples were taken by the Berlin Centre of Competence for Water during a sampling campaign after heavy rainfall events in 2016. Additional samples were drawn and analysed from a bathing water located at river 2 (river 2/bathing water) in the years 2018 and 2019. Water samples were processed directly after sampling or stored at − 80 °C until further processing.

### Sample Concentration

**Fig. 1:**
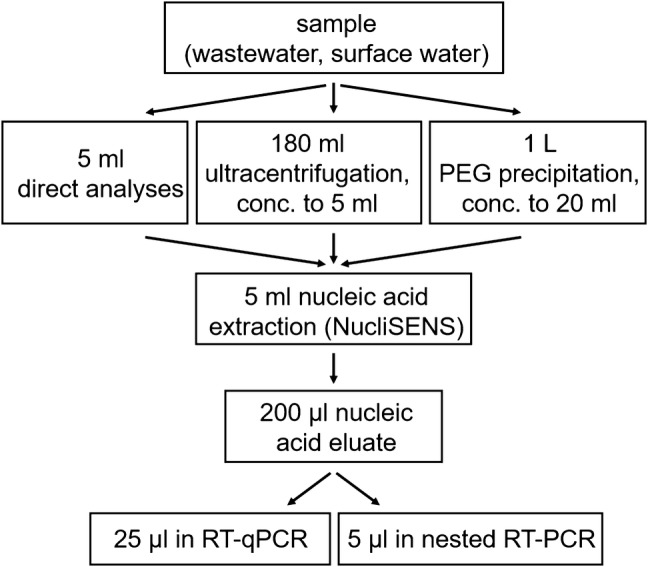
Flow chart of methods applied on wastewater and river water samples for HEV RNA detection

In environmental samples, human-pathogenic viruses are mostly present in low or very low concentrations and have to be further concentrated for analyses. In our study we used ultracentrifugation (U) and polyethylene glycol (PEG) precipitation (Fig. 1).

Ultracentrifugation was performed according to a previously described method (La Rosa et al. 2007). In brief, 180 ml supernatant after initial centrifugation at 3000×*g* for 10 min was pelleted by 2 h centrifugation at 160,000×*g* with a 45Ti rotor in an Optima L-100 K ultracentrifuge (Beckmann, Germany) and resuspended in 5 ml PBS for nucleic acid extraction.

For virus concentration by PEG precipitation (Manor et al. 2007), PEG 6000 (80 g) and NaCl (17.5 g) were added to 1 l water samples, mixed for 1 h and stored overnight at 4 °C. Subsequently, precipitates containing the viruses were collected after 1 h centrifugation at 12,200×*g*. Pellets were resuspended in 15 ml PBS, 15 ml chloroform (to destroy bacteria) and 150 µl Tween-80. After centrifugation for 15 min at 1400×*g*, the top layer was saved and the lower chloroform layer was removed. Remaining pellets were resuspended in 0.05 mol/l glycine pH 7.2 with 3% beef extract. Centrifugation was repeated and both supernatants were combined to a final volume of about 20 ml. Exact volumes were noted for calculating virus concentrations in the original samples.

### Nucleic Acid Extraction

Nucleic acid extraction was performed with 5 ml volumes of concentrated samplesor 5 ml volumes of samples without further virus concentration steps (direct samples). The NucliSENS® easyMAG® (bioMérieux, Germany) method allows simultaneous extraction of DNA and RNA with same efficiencies. To assess the extraction efficacy, a sample spiked with human adenovirus 2 with a defined concentration was included for each set of samples subjected to the nucleic acid extraction procedure. The method was used according to the manufacturer’s protocol, with slight modifications. Centrifugation was performed at 6000×*g* for 5 min after lysis buffer incubation to eliminate large disturbing particles present in turbid water samples. In addition, purified nucleic acids were eluted two times in 100 µl elution buffer resulting in a final volume of 200 µl to allow analyses of several qPCR reactions.

### HEV-Specific Quantitative Real-Time RT-PCR

Primers designed by Jothikumar et al. (2006) were used for quantitative RT-PCR. Probes were used either as described by Jothikumar et al. (2006) or in a modified version (Garson et al. 2012) using the quencher MGB (Minor groove binder) to reduce the risk of false negative real-time RT-PCR results. RT-qPCR was performed in a volume of 25 µl using the QuantiTect Probe RT-PCR Kit (QIAGEN, Germany) with a probe concentration of 0.2 µM. The following cycle conditions were applied: 30 min at 50 °C, 15 min at 95 °C and 45 cycles with 15 s at 94 °C and 1 min at 56 °C. Each reaction mix contained 10 µl of undiluted or 1:10 diluted templates (4 reactions per sample) to detect putative inhibition of the RT-qPCR reaction. Copy numbers are calculated based on all reactions, if undiluted and diluted samples correspond. In the case of partial inhibition in the undiluted samples, copy numbers of the diluted samples were chosen for quantification.

Double-stranded DNA Gene Strands (Eurofins Genomics, Sweden) containing the specific amplification sequence were applied as quantitative HEV standards in concentrations from 10^6^ HEV copies/10 µl to 10^1^ HEV copies/10 µl to generate a standard curve for determination of virus copy numbers in the samples. Standard deviations of samples during the 1-year surveillance were calculated from two to seven monthly samples. The calculation of the limit of detection (LOD) of HEV RNA was based on duplicates of 10 µl nucleic acid templates per RT-qPCR reaction with at least 1 HEV copy to be detected in the duplicate. If HEV RNA was not detected, the LOD concentration was used for further calculations. With 200 µl of viral nucleic acids eluted by the NucliSENS® easyMAG® method from 5 ml of direct water samples, the LOD was 200 copies/100 ml. Using nucleic acids from water samples concentrated by ultracentrifugation, an LOD of 6 copies/100 ml was achieved and the LOD of PEG-precipitated samples was four copies per 100 ml. The limit of quantification (LOQ) was set ten times higher than the LOD of each method.

### HEV-Specific Nested RT-PCR

Primers for nested RT-PCR, which amplify a 332 bp product from the HEV open reading frame 1 (ORF1) were designed by Johne et al. (2010). RNA from the HEV isolate 47832c (Johne et al. 2014) was used as positive control. For the first RT-PCR with a total reaction volume of 25 µl, 5 µl of template was amplified using the OneStep Ahead RT-PCR Kit (QIAGEN, Germany). Cycling profile included the following settings: 10 min at 50 °C, 5 min at 95 °C, 40 cycles of 10 s at 95 °C, 10 s at 55 °C, 10 s at 72 °C and 2 min at 72 °C. The second nested PCR was performed with 2 µl template from the first RT-PCR. The Taq DNA Polymerase Kit (QIAGEN, Germany) was used according to protocol in a total reaction volume of 50 µl. Primer concentrations were 0.3 µM and cycling conditions were the following: 3 min at 94 °C, 35 cycles of 45 s at 94 °C, 45 s at 60 °C, 1 min at 72 °C and 10 min at 72 °C.

PCR fragments were separated by gel electrophoresis on 1.5% agarose gels in 1 × TBE buffer with 10 µl of 10,000×*g* GelRed staining (Biotium, Germany) per 100 ml agarose solution. Loading buffer (Thermo Scientific, Germany) was mixed with the PCR products and gels were run for 50 min at 90 V. Low range DNA ladder (5 µl) was used as a size marker (Thermo Scientific, Germany).

### DNA Sequencing and Nucleotide Sequence Analyses

Bands of the expected length (332 bp) were excised and purified according to the protocol from innuPREP DOUBLEpure Kit (Analytik Jena, Germany). The cDNA was eluted twice in 30 µl elution buffer, combined and sequenced by Eurofins (Germany).

All HEV sequences determined in this study were submitted to NCBI GenBank under accession numbers MT087290 to MT087304. Sequence alignments and phylogenetic trees were constructed with Molecular Evolutionary Genetics Analysis Version 7.0 (MEGA 7) software (Kumar et al. 2016). The MUSCLE program was used for multiple sequence alignment and maximum likelihood as statistic method based on the Kimura 2-parameter model (Kimura, 1980). The phylogenetic trees were validated by replicating with 1000 bootstraps. Obtained HEV sequences were aligned to 41 HEV-subtype reference sequences (or a subset of 19 genotype 3 reference subtype sequences), as recommended by Smith et al. (2016b). In addition, sequences were aligned to the HEV-3c positive control (isolate 47832c from Johne et al. 2014) and 17 sequences from HEV infected patients from the Charité Hospital in Berlin (Wang et al. 2018a).

### Statistical Analyses

**Fig. 2:**
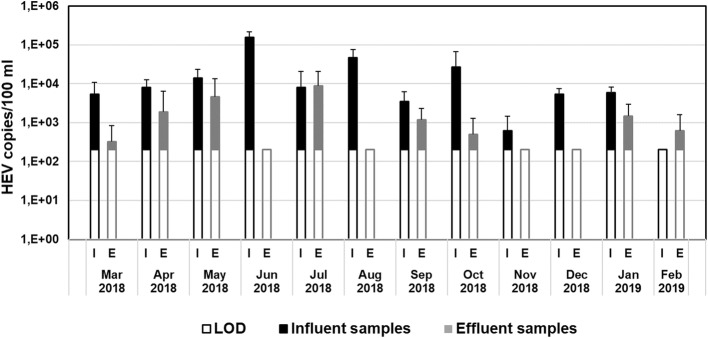
Comparison of HEV RNA concentrations in monthly influent samples (I) and effluent samples (E) of WWTP 1, analysed by RT-qPCR without virus concentration steps. Black and grey bars represent measured HEV concentrations above the LOD (open bars). LOD is the limit of detection with 200 copies/100 ml

**Fig. 3:**
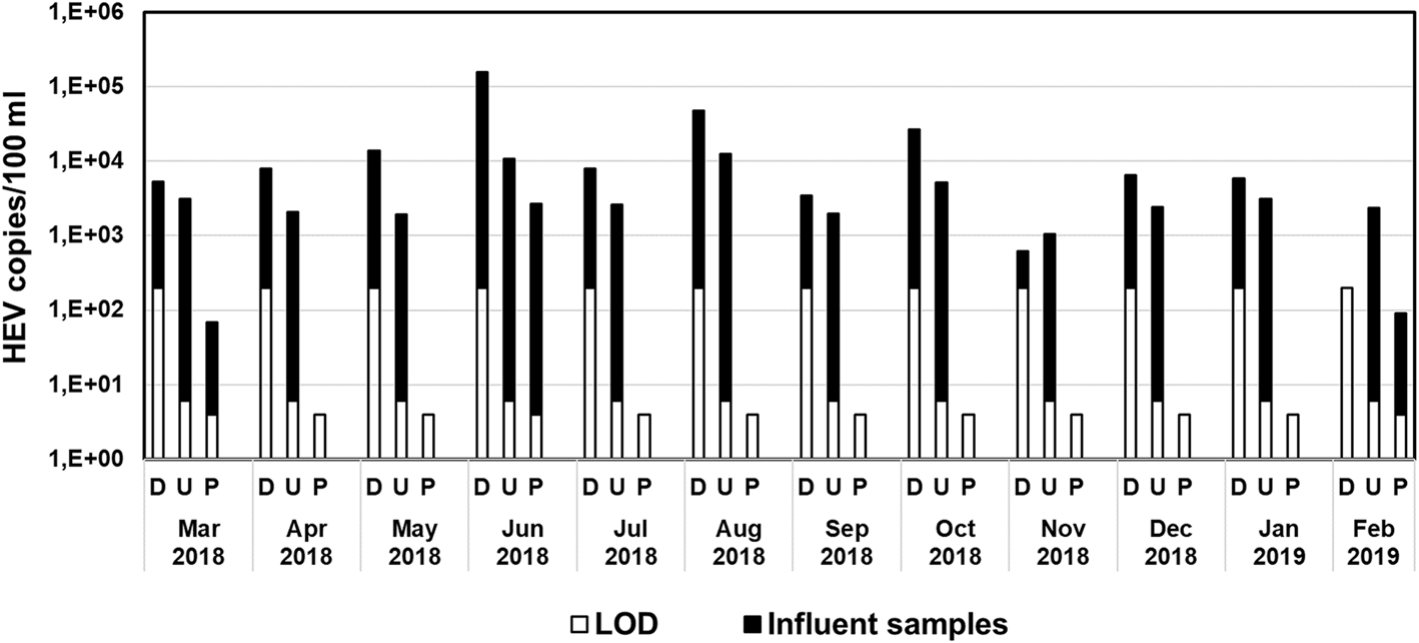
Concentration of HEV RNA in monthly influent samples of an urban WWTP over a period of one year. For comparison of sampling methods direct samples (D) and samples concentrated by ultracentrifugation (U) and PEG precipitation (P) were analysed by RT-qPCR. Black bars represent measured HEV concentrations above the LOD (open bars), which differ in each method

Statistical analyses were performed with Microsoft Excel. As quantification data are not normally distributed but ordinally scaled, the Mann–Whitney U test was used to determine the statistical significance at a 95% confidence level. This test was carried out to evaluate statistical differences between monthly virus concentrations of WWTP influent samples and WWTP effluent samples (Fig. 2) as well as between different virus concentrations methods (Fig. 3).

## Results

### Monitoring of HEV in Environmental Water Samples by RT-qPCR

Surface waters as well as influent and effluent wastewater samples of WWTPs from urban and suburban areas were monitored for HEV by RT-qPCR. Four urban and suburban WWTPs, differing in their catchment areas and cleaning capacities, were investigated (Table 1). Of 111 wastewater influent samples collected in the urban WWTP 1, 84% were positive for HEV RNA with a median concentration of 3 × 10^3^ copies/100 ml. The median concentration of all 111 tested samples was 2 × 10^3^ copies/100 ml.

**Table 1:** Detection of HEV RNA in WWTP influent and effluent samples by RT-qPCR

	Cleaning capacity [m^3^/day]	WWTP Influent samples				WWTP Effluent samples			
		Tested (n)	Positive [n/ (%)]	Median positive samples*	Median all samples*	Tested (n)	Positive [n/ (%)]	Median positive samples*	Median all samples*
WWTP 1	257.000	111	93 (84%)	3 × 10^3^	2 × 10^3^	83	26 (31%)	1 × 10^3^	< LOD
WWTP 2	5.500	10	9 (90%)	2 × 10^3^	1 × 10^3^	2	1 (50%)	8 × 10^2^	4 × 102
WWTP 3	119.000	7	6 (86%)	4 × 10^3^	3 × 10^3^	3	3 (100%)	4 × 10^2^	4 × 102
WWTP 4	40.000	6	6 (100%)	1 × 10^4^	1 × 10^4^	nt	nt	nt	nt

*WWTP* wastewater treatment plant, *LOD* limit of detection, *nt* not tested

*[HEV copies/100 ml]

HEV RNA was also detected in 26 out of 83 wastewater effluent samples (31%) of WWTP 1 with a median concentration of 1 × 10^3^ copies/100 ml in positive samples. However, the median concentration of all 83 tested effluent samples was below the limit of detection (LOD).

In three suburban WWTPs (WWTP 2–4), HEV RNA was detected in 86–100% of the influent samples with median concentrations of positive samples in the range of 2 × 10^3^ copies/100 ml–1 × 10^4^ copies/100 ml. Effluent samples of suburban WWTPs were positive for HEV RNA at rates of 50% (WWTP 2) and 100% (WWTP 3). Median concentrations of these positive samples were 8 × 10^2^ copies/100 ml and 4 × 10^2^ copies/100 ml, respectively. Median concentrations of all tested effluent samples of these suburban WWTPs were 4 × 10^2^ copies/100 ml.

The results for HEV monitoring of surface waters are shown in Table 2. About 30% of 90 tested samples of two urban rivers under normal weather conditions (river 1 and river 2) were positive for HEV RNA with median concentrations of 6 × 10^2^ copies/100 ml and 9 × 10^2^ copies/100 ml, respectively. Although effluents of WWTP 1 are released into river 2 about 3 miles upstream of the sampling site, median concentrations of all river samples were below the LOD. However, after heavy rainfall events, causing combined sewer overflows (CSOs) upstream into river 1 (river 1/CSO), 75% of the samples were positive for HEV RNA with a median concentration of 2 × 10^3^ copies/100 ml.

**Table 2:** Detection of HEV RNA in surface water samples by RT-qPCR

	Surface water samples			
	Tested (n)	Positive [n/(%)]	Median positive samples*	Median all samples*
River 1	21	7 (33%)	6 × 10^2^	< LOD
River 1/CSO	16	12 (75%)	2 × 10^3^	2 × 10^3^
River 2	69	21 (30%)	9 × 102	< LOD
River 2/BW	55	8 (15%)	3 × 102	< LOD

*LOD* limit of detection, *BW* bathing water, *CSO* combined sewer overflow

* [HEV copies/100 ml]

In a bathing water located at the urban river 2 (river 2/BW) downstream the first sampling site of river 2, only eight out of 55 samples (15%) were positive for HEV. The median concentration of eight positive samples of this bathing water was 3 × 10^2^ copies/100 ml, but the median concentration of all 55 tested samples was below the LOD.

### One-year HEV Surveillance of a Wastewater Treatment Plant

To investigate if the high variability in the concentrations of HEV in influent and effluent samples was affected by environmental or seasonal influences, the central urban WWTP 1 was surveilled during a complete cycle of a year (Fig. 2).

In WWTPs, virus concentrations are sufficiently high to be detected in small volumes without further virus concentration steps. Therefore, direct samples were measured from March 2018 to February 2019 in influent and effluent samples of WWTP 1. HEV RNA was detected in 11 out of 12 monthly influent samples and in eight out of 12 effluent samples based on the LOD of 200 copies/100 ml.

Influent and effluent samples were taken the same day without considering the passage time of wastewater treatment. Several samples were collected each month. The mean concentration of all monthly measured samples is shown in the figure for each month and was used to compare influent and effluent samples of WWTP 1.

The mean value of calculated HEV RNA concentrations of 12 monthly influent samples over the surveilled year was 2 × 10^4^ genome copies/100 ml. Effluent samples resulted in a mean of 2 × 10^3^ copies/100 ml over this one-year period. The average HEV RNA reduction during the passage of the WWTP was about 1 log_10_, comparing influent and effluent samples above the LOD. Moreover, HEV concentrations of influent samples are significantly higher than from effluent samples (Mann–Whitney U test, *p* < 0.05). With a limit of quantification (LOQ) set to tenfold LOD, 10 of 12 influent samples and only 2 effluent samples were positive for HEV RNA, demonstrating the clearing effect of at least 1 log_10_ in the wastewater treatment plant. During the surveilled year, no obvious seasonal pattern of HEV occurrence in wastewater samples was observed.

### Comparison of HEV Concentration Methods

To investigate if sample preparation methods have an impact on the detection rate and the measured HEV RNA concentrations, direct samples and samples concentrated by ultracentrifugation and polyethylene glycol precipitation were compared over a cycle of one year using influent samples of WWTP 1 (Fig. 3).

Each of these three methods has a different limit of detection, namely 4 copies/100 ml or 6 copies/100 ml for PEG precipitation and ultracentrifugation, respectively, or 200 copies/100 ml for direct samples. In direct wastewater influent samples, HEV RNA was detected in 11 out of 12 monthly samples. In samples concentrated by ultracentrifugation, HEV RNA was found each month. Calculated HEV concentrations in direct samples and samples concentrated by ultracentrifugation were in the similar range, in contrast to PEG-processed samples, which resulted in lower HEV RNA concentrations and lower detection rates. Using the PEG method, viruses were detected only three times during this surveillance year and thus they were clearly significantly different from direct samples and samples concentrated by ultracentrifugation (Mann–Whitney U test, *p* < 0.05).

### Genotyping of HEV from Environmental Water Samples

**Fig. 4:**
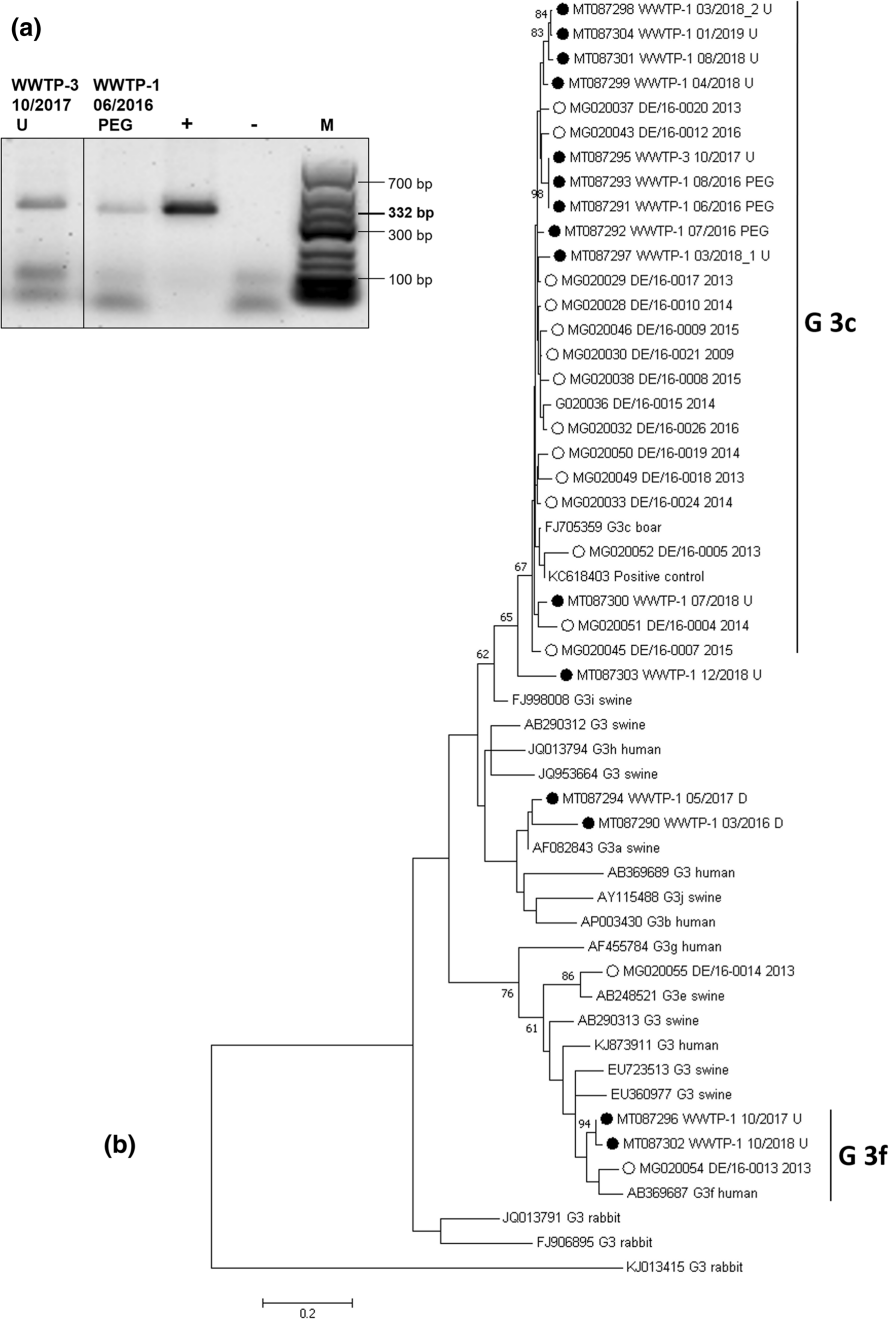
Characterization of HEV strains from wastewater samples by gel electrophoresis and sequencing. **a** Exemplary agarose gel with HEV positive samples (332 bp fragments) from two WWTP influent samples. **b** Maximum likelihood phylogenetic consensus tree of HEV strains detected in urban wastewaters. Numbers at the nodes represent bootstrap values > 60. Scale bar indicates the genetic distance (nucleotide substitutions per site). Identified HEV sequences detected in wastewater samples are marked with a black dot. Names consist of accession numbers, places, months, years of sampling and preparation methods (D: direct sample, U: ultracentrifugation, PEG: polyethylene glycol precipitation). Sequences from HEV infected patients are labelled with open dots. HEV sequences were aligned to 41 HEV-subtype reference sequences denoted by accession number, subgenotype and source of first detection. Since all identified sequences belonged to genotype HEV-3, only sequences of this genotype are shown. Three rabbit HEV-3 sequences were used as outgroup

To characterize the detected HEV strains in more detail, sequencings were carried out to identify HEV genotypes and subgenotypes in urban and suburban water samples (Fig. 4).

After performing nested RT-PCR with HEV-specific primers, wastewater influent samples of two different WWTPs (WWTP 1 and WWTP 3) clearly showed the characteristic 332 bp fragments. An exemplary agarose gel with amplified HEV nested RT-PCR products from HEV ORF1 is shown in Fig. 4a. Out of 173 tested wastewater influent samples, 15 samples (9%) displayed a clear band on the gel and fragments were subjected to sequencing. None of the 94 wastewater effluent samples and 57 river water samples showed a clear 332 bp band in the nested RT-PCR suitable for sequencing (data not shown).

Genotyping and subtyping were performed by sequence alignments with reference strains followed by phylogenetic analyses (Fig. 4b). All identified sequences belonged to HEV genotype 3. Therefore, only reference sequences of genotype 3 are shown. Bootstrap values > 60 are reported. HEV genotype 3c was the most prevalent subtype detected in10 wastewater influent samples. Two wastewater samples were identified as HEV genotype 3f. For three other samples no subtypes were classified. Of these 15 genotyped HEV strains from wastewater samples, ten were obtained from samples concentrated by ultracentrifugation, three from samples prepared by PEG precipitation and two from samples without further virus concentration steps. Moreover, the HEV genotypes identified in samples of urban and suburban WWTPs from the years 2016–2019 were compared to sequences of HEV infected patients from the same area from 2009–2016 (Wang et al. 2018a). As seen in the phylogenetic tree (Fig. 4), most of the wastewater and patient sequences cluster in subtype 3c or 3f.

## Discussion

This study presents a quantitative surveillance and genotyping of HEV strains in urban and suburban wastewater influent and effluent samples as well as in surface waters.

The zoonotic genotype 3 of HEV is autochthonous in many industrialized countries (Clemente-Casares et al. 2003; Meng 2010; Dalton et al. 2014). Besides foodborne transmission of this genotype, environmental transmission pathways have also been proposed. In the present study, a wide distribution of HEV RNA in environmental waters in Germany was identified, which may pose a risk of environmental transmission of HEV. However, HEV RNA detected by PCR methods does not necessarily represent intact and infective virus particles.

The highest detection rates (84–100%) of HEV RNA by quantitative PCR were found in wastewater influent samples, with a detection rate of 84% in WWTP 1 (Table 1). In contrast, only 31% of the effluent samples of WWTP 1 were positive for HEV RNA, demonstrating a cleaning effect of the WWTP with regard to HEV. In accordance with this finding, quantitative data on all tested samples of WWTPs indicate an HEV RNA reduction of about 1 log_10_ during treatment. This result was validated by the HEV surveillance of WWTP 1 over a complete one-year period, showing an average decrease from 2 × 10^4^ genome copies/100 ml in influent samples to 2 × 10^3^ copies/100 ml in effluent samples (Fig. 2). These effluent samples were taken before further UV treatment in the WWTPs. However, in summer, HEV RNA reduction during wastewater treatment is expected to be higher, since WWTP 1 is run during the bathing season with an additional UV treatment of effluents prior to release in surface waters.

Elimination of viruses in WWTPs depend on the characteristic features of the viruses as well as on the structures and combinations of treatment steps of the plants. Furthermore, there is a lack of data for HEV RNA reduction during treatment in WWTPs. So far, reports with quantified HEV concentrations in environmental waters are rare and mainly restricted to wastewater influent samples. The HEV concentrations in wastewater influents determined in this study correspond to the concentrations reported by Rodriguez-Manzano et al. (2010), Masclaux et al. (2013), Wang et al. (2018b) and Miura et al. (2016). In contrast to the findings of Masclaux et al. (2013), where HEV RNA was detected more frequently in summer, no clear seasonal pattern of HEV RNA occurrence was observed in the present study, similar to the report of Ram et al. (2016).

Since wastewater effluents are discharged into rivers, further investigations were carried out in two urban rivers (Table 2). Under normal weather conditions, about 30% of these river samples were positive with low concentrations of HEV RNA. However, the median HEV RNA concentration of all tested river samples was below the LOD. These low detection rates and low RNA concentrations are reasonable due to virus dilution in big water volumes. Similar results have been reported in Italian surface waters impacted by runoffs from grazing land and discharges from treatment plants, where 25% of the tested water samples were HEV RNA-positive (Idolo et al. 2013). In line with the results from the two rivers under normal conditions, only 15% of samples from a bathing water were positive for HEV RNA, with a median concentration of all tested samples below the LOD. Therefore, no evidence of an increased health risk was found at this bathing area. This reflects the water management efforts to maintain the bathing water quality under normal weather conditions.

In urban areas, mixed channels for sewage and rain water may reach capacity limits after heavy rainfall events and lead to release of uncleared wastewater into rivers (combined sewer overflows, CSOs).

Such CSOs seem to have a high impact on HEV detection rates and concentrations in rivers, as seen in this study for river 1, where several CSO sites are located. After three heavy rainfall events in summer 2016 causing CSOs in river 1, HEV positive samples increased from 33 to 75%, with a median copy number of 2 × 10^3^ copies/100 ml. Therefore, urban rivers may contain high HEV RNA concentrations during rainfall-affected periods, thereby increasing the public health risk of HEV infections over the faecal-oral route by bathing or recreational activities in the polluted urban rivers.

Detection and quantification of HEV RNA in environmental water samples is challenging. If low virus concentrations are present in large sample volumes the methods used for virus concentration can have significant influences. We therefore compared the detection rates obtained by ultracentrifugation and PEG precipitation, using samples with or without virus concentration steps (Fig. 3). Direct sampling and ultracentrifugation revealed comparable monthly detection rates, whereas the PEG-processed samples resulted in lower HEV RNA findings. Direct virus detection is easy to perform but since small volumes are used, the limit of detection is much higher than for methods with virus concentration steps. Due to the small tested volumes, calculated virus concentrations could be over- or underestimated. Therefore, when samples are tested by different methods, final virus concentration should always be reported together with the concentration method to ensure valid comparisons of the obtained data.

Ultracentrifugation and PEG precipitation are standard virus concentration methods for detection of HEV in sewage samples (Clemente-Casares et al. 2003; Rodriguez-Manzano et al. 2010; Masclaux et al. 2013; Myrmel et al. 2015; Ram et al. 2016; Iaconelli et al. 2017; Wang et al. 2018b; Matos et al. 2018). The 25% positive samples during the 1-year period using the PEG precipitation method were comparable to findings of Masclaux et al. (2013) and Miura et al. (2016), which reported rates of 32% and 22%, respectively.

In our hands, detection rates were much higher using the ultracentrifugation method. This method does not need any addition of chemicals and since viruses tend to attach to suspended matter (Jin and Flury 2002), ultracentrifugation is a suitable method for influent samples of WWTPs. Moreover, these ultracentrifugated samples were most suited for genotyping of HEV strains.

In contrast to quantitative RT-PCR detection, longer fragments need to be amplified for genotyping, which may result in a lower sensitivity. In the present study, 15 amplicons (9%) of the WWTP influent samples could be successfully sequenced. Using nested RT-PCR, similar results of 5–13.5% of influent samples positive for HEV RNA were reported from Italy and Spain (Rusiñol et al. 2015; Iaconelli et al. 2017; Alfonsi et al. 2018). For genotyping, samples with virus concentrations steps were most suitable, since 13 amplicons were sequenced from ultracentrifugated or PEG-precipitated samples. Although the nested primer system was able to amplify all HEV genotypes (Johne et al. 2010), only HEV-3 strains were detected in environmental water samples. The most prevalent HEV subtype was HEV-3c. In addition, two samples contained HEV genotype 3f. HEV-3c and HEV-3f were also recently reported in wastewater in Italy (Di Profio et al. 2019). Of our three samples which could not be subtyped exactly, two are most likely of subtype 3a and one of subtype 3c or 3i.

The detected environmental HEV genotypes correlate well with reported subgenotype data from clinical samples from Germany (Vollmer et al. 2012; Tabatabai et al. 2014; Adlhoch et al. 2016). Genotype HEV-3c was reported to be the most prevalent genotype in German blood donors, and genotypes 3a and 3e were also found in clinical samples (Vollmer et al. 2012). Moreover, HEV-3c was identified in the first German clinical report of acute hepatitis E during pregnancy (Tabatabai et al. 2014) and is the most common type in the European Union/ European Economic Area (EFSA 2017).

Most of the wastewater influent samples were obtained from an urban WWTP with a catchment area of about 1.1 million people of the Berlin area. A recent study investigated HEV genotype 3 variants in patients from the same area and identified subtype 3c as the most prevalent HEV strain, besides genotypes 3e and 3f (Wang et al. 2018a). In our study, subtype HEV 3c was also detected in the suburban WWTP 3 with a pig farm located nearby. However, the overall HEV detection rate did not differ from the other WWTPs. Besides domestic pigs, in which HEV infection is highly prevalent (Fernández-Barredo et al. 2007; Jiménez de Oya et al. 2011; Dremsek et al. 2013), wild boars have been identified as a possible source of HEV RNA. In addition, it has to be considered that Berlin also harbours a large population of wild boars (Stillfried et al. 2017). In a current study, a high degree of nucleotide sequence homology in a wild boar isolate and a human isolate was detected and zoonotic HEV-3c and 3a were identified in wild boars in the Berlin/Potsdam area (Schielke et al. 2009), corresponding with the detection of HEV-3c and 3a RNA in this study. In cities, HEV transmission from wild boars to humans has to be taken into consideration, either by direct or by indirect transmission by surface waters, environment, or other carrier animals (Schielke et al. 2009).

The composition of subtypes detected in the environmental waters in the present study reflects the circulation of HEV strains in humans and animals in the same region. Previous reports from other countries also demonstrated a correlation between detected viruses in wastewater and clinical cases (Hellmér et al. 2014; Ivanova et al. 2019).

Detection of viruses in environmental waters can contribute to a better understanding of the epidemiology and prevalent strains in the population. Although foodborne transmission is considered as the main pathway of zoonotic HEV infection, environmental transmission should not be neglected and water monitoring should be integrated in the One Health approach to reduce public health risks.
